# Characterization of Acellular Cartilage Matrix-Sodium Alginate Scaffolds in Various Proportions

**DOI:** 10.1089/ten.tec.2023.0348

**Published:** 2024-04-03

**Authors:** Wang Lu, Mengchu Yang, Yanan Zhang, Baoxi Meng, Fulian Ma, Wanjun Wang, Teng Guo

**Affiliations:** ^1^ Department of Plastic Surgery and The Second Affiliated Hospital of Zhengzhou University, Zhengzhou, Henan, China.; ^2^ Department of Pharmacy, The Second Affiliated Hospital of Zhengzhou University, Zhengzhou, Henan, China.

**Keywords:** acellular cartilage matrix, sodium alginate, scaffolds, adipose-derived stem cell, 3D bioprinting, rabbit

## Abstract

The development of three-dimensional (3D) bioprinting technology has provided a new solution to address the shortage of donors, multiple surgeries, and aesthetic concerns in microtia reconstruction surgery. The production of bioinks is the most critical aspect of 3D bioprinting. Acellular cartilage matrix (ACM) and sodium alginate (SA) are commonly used 3D bioprinting materials, and there have been reports of their combined use. However, there is a lack of comprehensive evaluations on ACM-SA scaffolds with different proportions. In this study, bioinks were prepared by mixing different proportions of decellularized rabbit ear cartilage powder and SA and then printed using 3D bioprinting technology and crosslinked with calcium ions to fabricate scaffolds. The physical properties, biocompatibility, and toxicity of ACM-SA scaffolds with different proportions were compared. The adhesion and proliferation of rabbit adipose-derived stem cells on ACM-SA scaffolds of different proportions, as well as the secretion of Collagen Type II, were evaluated under an adipose-derived stem cell chondrogenic induction medium. The following conclusions were drawn: when the proportion of SA in the ACM-SA scaffolds was <30%, the printed structure failed to form. The ACM-SA scaffolds in proportions from 1:9 to 6:4 showed no significant cytotoxicity, among which the 5:5 proportion of ACM-SA scaffold was superior in terms of adhesiveness and promoting cell proliferation and differentiation. Although a higher proportion of SA can provide greater mechanical strength, it also significantly increases the swelling ratio and reduces cell proliferation capabilities. Overall, the 5:5 proportion of ACM-SA scaffold demonstrated a more desirable biological and physical performance.

**Impact statement:**

This study provides a more comprehensive *in vitro* assessment of material selection for bioinks in three-dimensional (3D) bioprinting technology for microtia reconstruction. By evaluating the biological performance and physical properties of acellular cartilage matrix-sodium alginate (ACM-SA) scaffolds in different proportions, we found that the 5:5 proportion of ACM-SA scaffold is more suitable as a constituent material for 3D bioprinting compared to other proportions. This provides meaningful foundational research data for our further *in vivo* animal experiments involving 3D bioprinted scaffolds loaded with cells.

## Introduction

Microtia is one of the more common types of congenital craniofacial anomalies.^1^ The incidence rate of microtia in newborns in China is ∼3.06 per 10,000.^2^ In addition to varying degrees of auricular deformities, microtia commonly presents with external auditory canal atresia and underdeveloped middle ear structures. Currently, the main treatment for microtia is autologous costal cartilage (ACC) grafting. The advantage of this approach is the use of the patient's tissue, which has a low tissue rejection rate. However, due to the young age of the affected children, the source of donor tissue is limited, and typically they have to wait until the age of 6 for the surgery. Prolonged waiting periods can have implications for both the physical and psychological well-being of the children.^3^ In addition, the symmetry and aesthetics of the auricle rely on the experience and skills of the surgeon.^4^

ACC treatment for microtia requires multiple surgeries, which can cause localized pain and create a sense of fear in the affected children due to the multiple procedures. 3D bioprinting offers a more optimal treatment option for patients with microtia, providing improved outcomes.^5–10^ 3D bioprinting reduces the number of surgical procedures required and eliminates the need to harvest ACC, thereby reducing the pain associated with surgery. By utilizing Computed Tomography (CT) reconstruction and 3D bioprinting of ear cartilage scaffolds, the issues of symmetry and aesthetics can be addressed.^1,11–14^ Moreover, this approach enables the reconstruction to be performed as early as 3 years of age, allowing for earlier intervention.

3D bioprinting is defined as the process of printing structures using living cells, biomaterials, and biomolecules.^15–17^ 3D bioprinting can provide patient-specific spatial geometries, control over microstructures, and the placement of different cell types for the manufacture of tissue engineering scaffolds. The choice of biomaterials plays a crucial role in the biologic properties (such as cell adhesion, proliferation, differentiation, and migration) and mechanical performance of the biologic scaffolds.^15,18–20^ Currently, a major research challenge in 3D bioprinting remains the selection and optimization of materials to ensure good printability, mechanical stability, biocompatibility, biodegradability, nontoxicity, high availability, and high shape fidelity after printing.^21–23^ Acellular cartilage matrix (ACM) derived from rabbit ear cartilage has been widely utilized due to its abundant source, excellent biocompatibility, and ability to promote stem cell proliferation.^24^

However, its low mechanical strength and poor printability have limited its application in 3D bioprinting. Sodium alginate (SA), a natural polysaccharide with a linear structure, finds extensive use in the field of biopharmaceuticals.^25^ It possesses favorable biocompatibility and degradability.^26^ When crosslinked with Ca2+, it can enhance the mechanical strength of the ACM. The characteristics of these two materials complement each other. In this experiment, bioinks were prepared by mixing ACM and SA in different ratios. Extrusion-based 3D printing technology and Ca2+ crosslinking were used to fabricate ACM-SA scaffolds. The physical properties of scaffolds with varying ratios were assessed, and their biocompatibility, as well as their impact on the chondrogenic differentiation and proliferation of rabbit adipose-derived stem cells (rADSCs), were evaluated through coculture. This experiment was conducted to provide reliable evidence for the application of ACM-SA scaffolds in low-load-bearing cartilage, such as ear and nasal cartilage.

## Methods and Experiment

### Materials and animals

All animal experiments were conducted in accordance with the Chinese Guidelines for Animal Welfare and Ethics, and received approval from the Animal Ethics Committee of Zhengzhou University (Reference No: 2022189). Unless specifically stated, the reagents used in the experiments were sourced from Beijing Solarbio Science & Technology Co., Ltd. For the following experiments, unless specifically stated otherwise, each group consisted of three samples.

### Morphological observation of ACM and DNA quantification analysis of ACM

Two New Zealand white rabbits were selected and euthanized by intraperitoneal injection of pentobarbital (90 mg/kg) to induce an overdose of anesthesia. Under sterile conditions, ear cartilage was isolated, and the connective tissue on the cartilage was removed. The cartilage samples were pretreated to a size of 2 mm^3^. They were immersed in liquid nitrogen for 15 min and then allowed to thaw at room temperature for 15 min. This freeze-thaw process was repeated six times. Then, the samples were placed in an ultrasonic cell disruptor (Ningbo Xinzhi Biotechnology Co., Ltd.) and operated at high power for 30 s, with three repetitions. They were washed with deionized water three times for 30 min each, followed by centrifugation at 500 *g* for 10 min to discard the supernatant.

The samples were frozen at −80°C for 6 h and immediately subjected to freeze-drying for 48 h at low temperatures. The cartilage pieces were soaked in a 4% sodium deoxycholate solution at a ratio of 1:100 (v/v) and incubated at 4°C with constant shaking for 24 h. Subsequently, the samples were washed three times with a phosphate-buffered saline (PBS) solution. DNase/RNase enzyme working solution was added at a ratio of 1:20 (v/v), and the samples were incubated at 37°C on a shaking incubator at a speed of 60 rpm for 12 h. The supernatant was discarded, and the samples were washed three times with sterilized deionized water. The remaining samples were placed in a −80°C freezer for 24 h and then removed. They were immediately transferred to a vacuum freeze-dryer (Beijing Boyikang Co., Ltd.) and subjected to freeze-drying for 24 h.

Subsequently, the dried cartilage pieces were ground using a low-temperature high-speed tissue grinder (Wuhan Seville Biotechnology Co., Ltd.). The resulting powder was sieved multiple times through a 200-mesh sieve and sterilized with ultraviolet irradiation for 24 h. Finally, the samples were stored at −20°C for future use.

Genomic DNA from both untreated natural rabbit ear cartilage tissue and decellularized ear cartilage tissue was extracted using a genomic DNA preparation kit (Merck & Co.). The concentration of DNA was measured using a UV spectrophotometer (Thermo Fisher Scientific Inc.), with the untreated natural rabbit ear cartilage tissue serving as the control group.

### Morphological observation of ACM

Using the Cell block Preparation Kit (Kunming Donghuan Technology Co., Ltd.), 1 g of ACM powder was placed into a standard centrifuge tube. Ten milliliters of cell fixative solution was added to the tube, and the mixture was fixed for 10 min. The centrifugation was performed at a speed of 550 *g* for 5 min. After centrifugation, the supernatant was discarded, and the precipitated powder was removed. The extracted powder was then added to the special centrifuge tube (with Matrigel) provided in the kit. Following the instructions, the special centrifuge tube was placed in a water bath, and heated at 85°C for 15 min, allowing the matrix and powder in the tube to mix thoroughly.

After removing the tube and centrifuging at 500 *g* for 15 min, the matrix inside the tube solidified again. The solidified block containing ACM powder was then removed, dehydrated successively, embedded in paraffin, and sectioned using a microtome (REM-3, Yamato Koki, Saitama, Japan) to produce slices (710 μm thick). These sections were stained using Hematoxylin and Eosin (H&E) and observed under a microscope. The ACM particles were fixed, freeze-dried, gold-sputtered, and observed by scanning electron microscopy (SEM).

### Preparation of biomaterial scaffold by mixing SA and ACM at different ratios

Separately, ACM powder and SA powder were weighed and mixed in ratios of 1:9, 2:8, 3:7 4:6, 5:5, 6:4, 7:3, 8:2, and 9:1. Each mixture was added to 100 mL of PBS solution and stirred at a constant temperature using a magnetic stirrer for 2 h to prepare a 4% ACM-SA solution. The solution was then left at room temperature for 12 h to allow the removal of bubbles. Subsequently, the prepared solution was printed using an extrusion-based 3D printer (Guangzhou Maipu Regenerative Medicine Technology Co., Ltd.). The scaffold design, including parameters such as diameter, height, fill density, and printing speed, was created using the built-in design software of the 3D printer. The slicing software provided by the 3D printer was used for slicing.

The solution was drawn into a 1 mL medical syringe and slowly extruded onto the low-temperature freezing platform (Thermo Fisher Scientific Inc.) of the 3D printer using a 27G needle. Upon contact with the freezing platform, the solution solidified into ice. The printed scaffold was then quickly transferred to a low-temperature 2% CaCl_2_ solution, where the carboxyl groups of SA chelated with Ca2+ ions to form a gel. After 5 min of crosslinking, the gel was transferred to deionized water to remove excess Ca2+ ions. After drying in a freeze-dryer for 48 h, the microstructure of the scaffold was observed using a SEM (Carl Zeiss AG).

The specific printing parameters were as follows: fill density 100%, printing material: ACM-SA, diameter 8 mm, height 2 mm, layer height 275 μm, nozzle movement speed 40 mm/s, maximum printing speed 80 mm/s, maximum nozzle extrusion speed 40 mm^3^/s, first layer printing speed 60 mm/s, and filling angle 90°. The scaffolds from each group were scanned and observed using an SEM, following the method described in section “Morphological observation of ACM.”

### Comparison of mechanical properties of ACM-SA scaffolds at different ratios

#### Porosity measurement of ACM-SA bioscaffold

We freeze-dried each group of scaffolds in a freeze dryer at −80°C for 48 h. Next, we placed the scaffold in a 15 mL centrifuge tube filled with anhydrous ethanol. We recorded the initial volume of the ethanol as V1. Then, we applied vacuum pressure until no more bubbles overflowed, and we recorded the volume at this point as V2. After removing the scaffold, we recorded the final volume as V3. We calculated the porosity (P) using the following formula:

(1) $$P=\left(V1-V3\right)∕\left(V2-V3\right)\times 100\%.$$

#### Degradation performance assessment

Each group of ACM-SA bioscaffolds was placed in Dulbecco's modified Eagle medium (DMEM) complete culture medium and incubated in a temperature-controlled shaking incubator at 37°C with low-speed agitation for 28 days. Weekly, the scaffolds were removed, washed twice with PBS solution, and then subjected to freeze-drying. We recorded the weight of the freeze-dried scaffolds and plotted a biodegradation curve. We recorded the dry weight of the scaffolds after freeze-drying as W1 and recorded the initial dry weight as W0. The degradation percentage (DP) was calculated using the formula:

(2) $$DP=\left(W0-W1\right)∕W0\times 100\%.$$

#### Swelling percentage of ACM-SA scaffolds

The freeze-dried ACM-SA scaffolds were placed in PBS, fully immersed for about 10 s, then removed and weighed, recorded as m3. The scaffolding was then weighed at 2 h intervals during dynamic treatment, and weights were recorded as m4 until no weight changes were observed. Using the provided formula, we plotted the swelling percentage (SW) curve of the scaffolds.

(3) SW=(m4−m3)∕m3×100%

#### Mechanical Properties Testing of ACM-SA scaffolds

The compression-unloading cyclic test of different scaffolds was conducted using an electronic universal testing machine (UTM2503). To replicate the actual mechanical properties of the scaffolds after cell seeding, we presoaked all scaffolds in PBS at 37°C for 24 h before the compression testing. The compression test was performed at a speed of 0.5 mm/min to a maximum strain of 80%. Based on the test results, the compressive modulus was calculated. Three samples were tested for each group of samples.

### rADSCs were cultured *in vitro*

After anesthetizing and disinfecting the rabbit, adipose tissue was obtained from the inguinal region of a 2-month-old female New Zealand white rabbit. We followed the method published by Liu et al.^27^ For the extraction and culture of rabbit rADSCs. The cells were cultured in a complete DMEM medium, which consisted of 88% DMEM culture medium, 10% fetal bovine serum, and 2% penicillin-streptomycin. The cultures were maintained at 37°C in a 5% carbon dioxide (CO_2_) cell culture incubator (Ashwell Cell Technology Co., Ltd.). The cells were subcultured at a ratio of 1:3.

### Phenotypic identification of rADSCs

Flow cytometry (Becton, Dickinson and Company) was used to perform cell immunophenotyping (CD29/CD44) on third-passage rADSCs. The procedure was as follows: Two samples were taken for each group and labeled with respective isotype antibodies as negative controls and test samples. Approximately 1 × 10^6^ cells in 100 μL were added to each test tube. In the isotype control tube, fluorescent-labeled monoclonal antibodies CD29 and CD44 were added as negative controls. In the test tube, fluorescent-labeled monoclonal antibodies CD29-PE and CD44-PE (Sigma-Aldrich Corporation) were added as experimental samples, 20 μL each, and mixed well. The tubes were incubated in the dark at room temperature for 30 min, followed by the addition of 2 mL of PBS and centrifugation at 1500 rpm for 10 min.

The supernatant was discarded, and 0.5 mL of fixation solution (staining buffer) was added. The isotype control group was measured first, followed by the experimental group. Data were acquired using software, and the proportion of positive cells was analyzed.

### Induction and validation of adipose-derived stem cell differentiation into chondrocytes

The third passage of rADSCs cells was selected and a single-cell suspension was prepared at a concentration of 2 × 10^6^ cells/mL. Of the rADSCs single-cell suspension, 0.5 mL was taken and centrifuged at 300 *g* for 5 min. After discarding the supernatant, 2 mL of adipose-derived stem cell chondrogenic induction medium was added. After mixing, the suspension was added to a six-well plate for culturing. The cells were cultured at 37°C and 5% CO_2_ in a constant temperature incubator, with medium change every 2 days. On the 14th day, the cells were fixed with 4% paraformaldehyde and stained with Alcian Blue Solution (pH 2.5). The cells were then observed under a microscope.

### Biotoxicity evaluation of ACM-SA scaffold

The biotoxicity of the scaffolds was evaluated using the soaking extraction method. We selected experimental samples with ACM: SA ratios of 1:9 and 6:4. The two groups of scaffolds were fully immersed in DMEM complete culture medium and incubated in a 37°C, 5% CO_2_ cell culture incubator for 24 h. Third-generation rADSCs were selected and adjusted to a cell density of 2 × 10^4^ cells/mL. For each group, 100 μL of the cell suspension was seeded into a 96-well plate, with a total of 9 wells divided into 3 groups: α, β, and γ. Group α served as a blank control group, where 100 μL of the DMEM complete culture medium was added. For groups β and γ, 100 μL of the extraction fluid with ACM:SA ratios of 1:9 and 6:4, respectively, was added.

Each group had 12 replicate wells. The medium in group α was changed every 48 h with DMEM complete culture medium, and for groups β and γ, the scaffold extraction fluid was changed each time. On days 1, 3, 5, and 7, 100 μL of fresh DMEM complete culture medium and 10 mL of CCK-8 reagent were added to each well. After incubation for 2 h, the absorbance (optical density, [OD] value) at 450 nm of each well was measured using an enzyme-linked immunosorbent assay (ELISA) reader (Shandong Wanxiang Environmental Technology Co., Ltd.).

### Assessment of adhesion and proliferation of rADSCs on ACM-SA scaffold under chondrogenic induction medium

The scaffolds were immersed in a DMEM culture medium for 24 h to achieve swelling equilibrium. After reaching equilibrium, we cut the scaffolds into dimensions of 5 × 5 × 2 mm. Each group of scaffolds was placed in a 24-well plate with three parallel samples per group. We selected the third generation of rADSCs and adjusted the cell concentration to 2 × 10^6^ cells/mL. We added 150 μL of single-cell suspension of rADSCs to each well and incubated them at 37°C and 5% CO_2_ in a constant temperature incubator for 24 h. After removing the 24-well plate, we added 1.5 mL of adipose-derived stem cell chondrogenic induction medium to each experimental well. The culture medium was changed every 2 days. On the third and seventh days after adding the induction medium, we used Calcein-AM/PI staining to observe cell adhesion and proliferation.

After culturing for 3 weeks, we removed the scaffolds, fixed the scaffold specimens, then embedded them in paraffin and sectioned. After dewaxing, hydration, and washing, we stained the cells with Alcian Blue staining solution (pH 2.5) for 30 min, washed twice with PBS, dehydrated, and sealed the slides. Finally, we observed the stained cells under an inverted microscope.

### Evaluation of collagen type II by rADSCs on ACM-SA scaffold under chondrogenic induction medium

As described in Section “Assessment of adhesion and proliferation of rADSCs on ACM-SA scaffold under chondrogenic induction medium”, we collected the culture medium from ACM-SA scaffolds at various time points (days 3, 5, 7, 9, 11, 14) to determine the levels of free Collagen Type II (COL-II) using an ELISA (Pel-Freez, LLC.). All reagents and samples were preequilibrated at room temperature. We added 100 μL of sample to the wells of an ELISA plate and incubated overnight at 4°C with gentle shaking. After incubation, the liquid was discarded and the wells were washed four times with PBS. The wells were then dried by inverting and tapping the plate to remove residual wash solution.

Following the assay kit's instructions, 50 μL of stop solution (I) was added to each well. The absorbance (OD value) at 450 nm was measured immediately using an ELISA reader. Three samples were prepared for each scaffold at each time point, and each sample was measured three times. After the measurements, the mean and variance were calculated.

### Statistical analysis

Quantitative data were obtained from a minimum of three independent experiments and are expressed as means ± standard deviation. After confirming the normal distribution of the data, statistical significance was assessed using Student's *t*-test or one-way analysis of variance with GraphPad Prism 8.0 software. A significance level of *p* < 0.05 was considered statistically significant.

## Results

### The structure of ACM

**FIG. 1.**
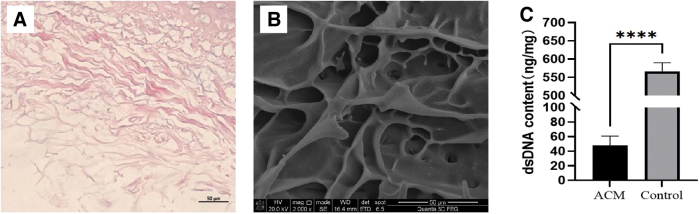
**(A)** H&E staining demonstrating successful decellularization of ACM, indicating absence of cell nuclei and preservation of extracellular matrix. **(B)** SEM image of ACM, showcasing a mesh-like collagen matrix structure conducive for adipose-derived stem cell growth. **(C)** Comparison of dsDNA Content in ACM versus Control. Statistical significance is indicated by ****p < 0.0001. ACM, acellular cartilage matrix; H&E, hematoxylin and eosin; SEM, scanning electron microscopy. Color images are available online.

The results of H&E staining and SEM image (Fig. 1A, B) confirm the successful decellularization of the cartilage slices. Moreover, compared with the natural rabbit auricular cartilage, the DNA content in the ACM group significantly decreased, from 562.5 ± 23.7 to 48.0 ± 12.8 ng/mg (Fig. 1C). The absence of cell nuclei and preservation of the extracellular matrix (ECM) indicate the effectiveness of the decellularization process. The collagen matrix forms a mesh-like structure that provides an ideal environment for the growth of rADSCs.

### The physical and mechanical characteristics of the ACM-SA scaffold

**FIG. 2.**
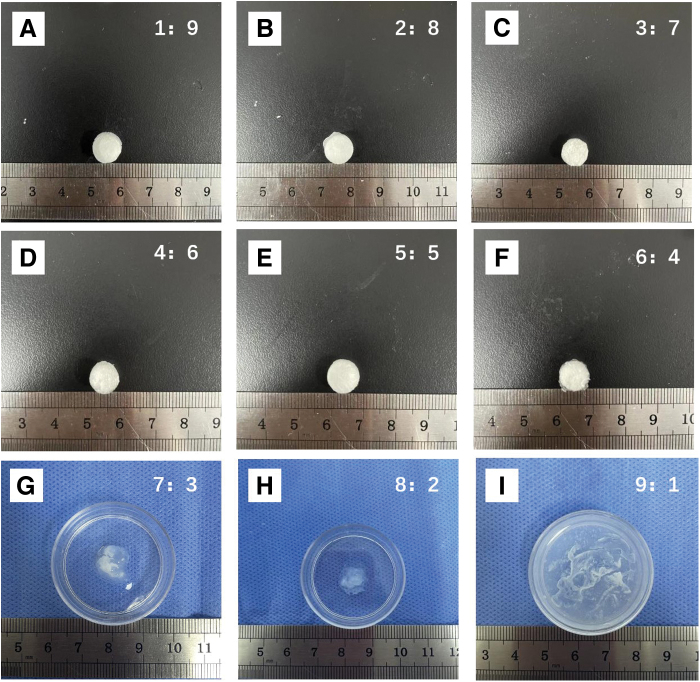
ACM-SA scaffolds formed by 3D printing and crosslinking with CaCl_2_. Ratios from 1:9 to 9:1 depicted in images **(A)** to **(I)**, showcasing successful scaffold formation in ratios 1:9 to 6:4, and shape failure in ratios 7:3 to 9:1. 3D, three-dimensional; SA, sodium alginate. Color images are available online.

After 3D printing and crosslinking with CaCl_2_, the ACM-SA bioink with ratios ranging from 1:9 to 6:4 successfully formed scaffolds (Fig. 2A–F). However, the bioinks with ratios ranging from 7:3 to 9:1 failed to maintain their shape (Fig. 2G–I).

#### The porosity of the ACM-SA scaffold

**FIG. 3.**
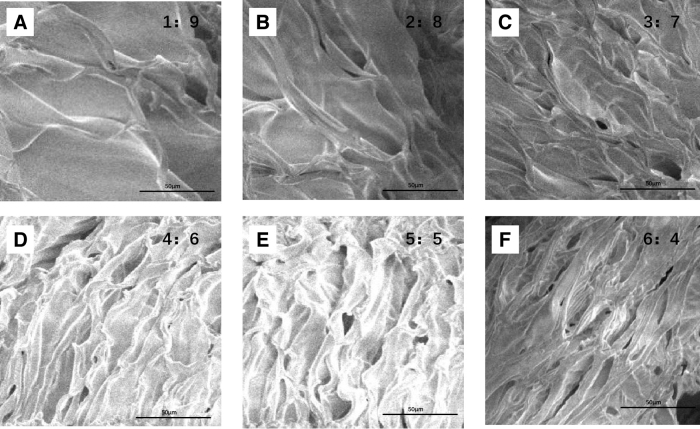
SEM images of ACM-SA scaffolds with varying ACM:SA ratios from 1:9 to 6:4. **(A)** Scaffold with an ACM:SA ratio of 1:9, showing the highest porosity. **(B)** Scaffold with an ACM:SA ratio of 2:8. **(C)** Scaffold with an ACM:SA ratio of 3:7. **(D)** Scaffold with an ACM:SA ratio of 4:6. **(E)** Scaffold with an ACM:SA ratio of 5:5. **(F)** Scaffold with an ACM:SA ratio of 6:4, demonstrating the lowest porosity as SA content increases.

The average porosity values of the ACM-SA scaffolds in each group were as follows: 88.63 ± 7.23%, 85.92 ± 4.47%, 80.48 ± 6.53%, 76.66 ± 8.61%, 75.04 ± 6.19%, and 63.24 ± 5.33%. A decreasing trend in porosity was observed with increasing SA content. The results obtained from SEM analysis were consistent with the porosity estimations calculated using anhydrous ethanol (Fig. 3).

#### The degradation percentage of the ACM-SA scaffold

**FIG. 4.**
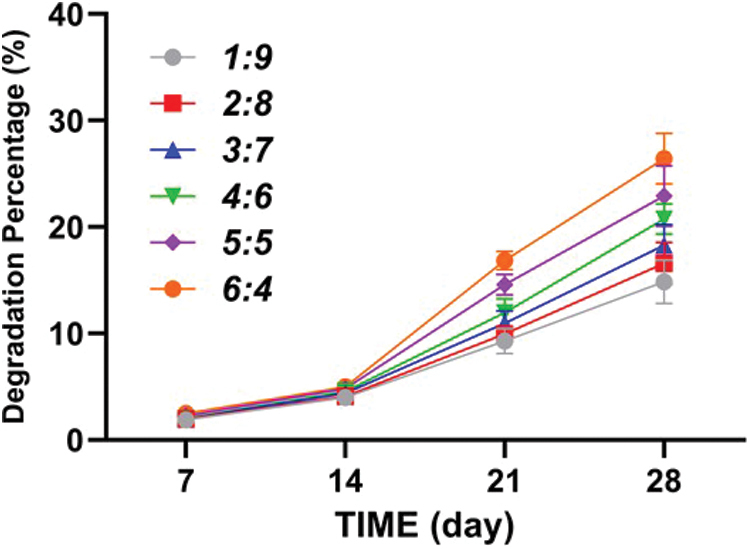
The degradation percentages of ACM-SA scaffolds with ratios from 1:9 to 6:4, illustrating a gradual increase in degradation rate over 28 days. Color images are available online.

As shown in Figure 4, ACM-SA scaffolds with ratios ranging from 1:9 to 6:4 exhibited a relatively low rate from day 1 to day 14. Following this period, the degradation rate increased significantly, as indicated in the referenced figure. By day 28, the degradation percentages of the scaffolds in each respective group were recorded at 14.93 ± 2.03%, 16.63 ± 1.97%, 18.36 ± 1.94%, 20.87 ± 1.42%, 23.03 ± 2.86%, and 26.53 ± 2.37%.

#### The swelling percentage of the ACM-SA scaffold

**FIG. 5.**
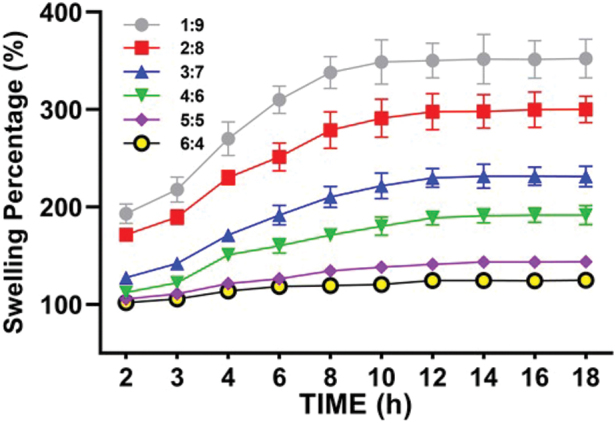
The swelling percentages of ACM-SA scaffolds across ratios from 1:9 to 6:4. Color images are available online.

As shown in Figure 5, the swelling percentage for the scaffolds in the ratios of 1:9 to 6:4 were 352.35 ± 19.75%, 300.10 ± 13.43%, 231.12 ± 10.57%, 191.50 ± 9.59%, 143.74 ± 5.12%, and 124.83% ± 3.13%, respectively. It is evident that as the SA content decreases, the swelling percentage gradually decreases as well. All groups of scaffolds exhibited minimal mass changes within the 12 to the 14 h range.

#### The mechanical properties of the ACM-SA scaffold

**FIG. 6.**
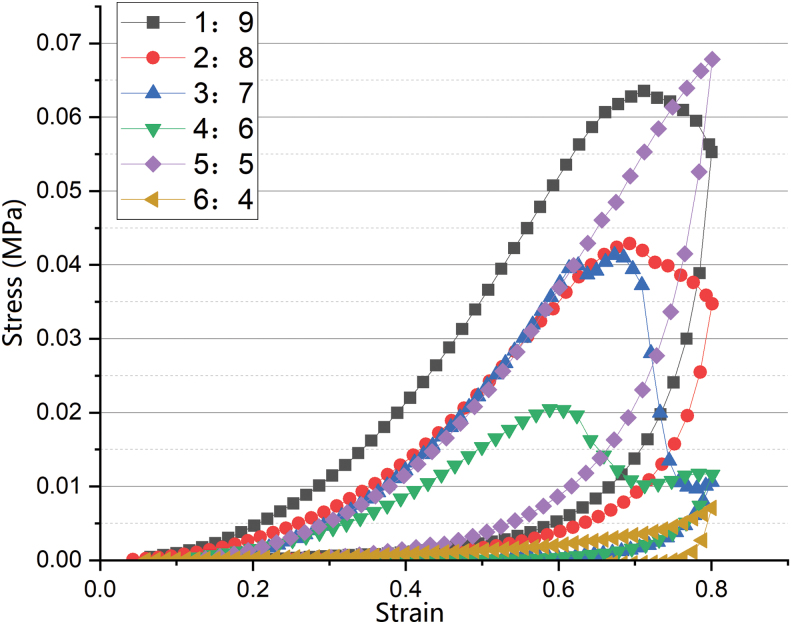
Stress-strain response curves for ACM-SA scaffolds with ratios from 1:9 to 6:4, demonstrating varying mechanical properties and compressive modulus. Color images are available online.

The compressive modulus of the ACM-SA scaffolds with ratios of 1:9, 2:8, 3:7, 4:6, 5:5, and 6:4 at a deformation of 50% were, respectively, 35.26 ± 1.12, 24.30 ± 0.80, 21.1 ± 0.90, 18.54 ± 0.59, 3.06 ± 0.13, and 2.00 ± 0.31 kPa. Upon reaching a strain of 60%, the 5:5 and 6:4 scaffolds did not exhibit a sudden drop in the stress-strain curve, unlike the other scaffolds which gradually deformed or fractured (Fig. 6). The maximum compressive strength of the ACM-SA scaffolds in the ratios of 1:9, 2:8, 3:7, and 4:6 was measured to be 63.57 ± 5.24, 42.88 ± 4.41, 41.33 ± 6.46, and 20.37 ± 3.12 kPa.

In addition, the compression test diagram indicates that the area enclosed by the loading/unloading curve in each group represents the dissipated energy per unit volume of the material. As the SA content decreases, the dissipated energy decreases while the scaffold's elasticity increases, thereby enhancing its recoverability after undergoing microdeformation.

### Validation of rADSCs and their differentiation into chondrocytes

**FIG. 7.**
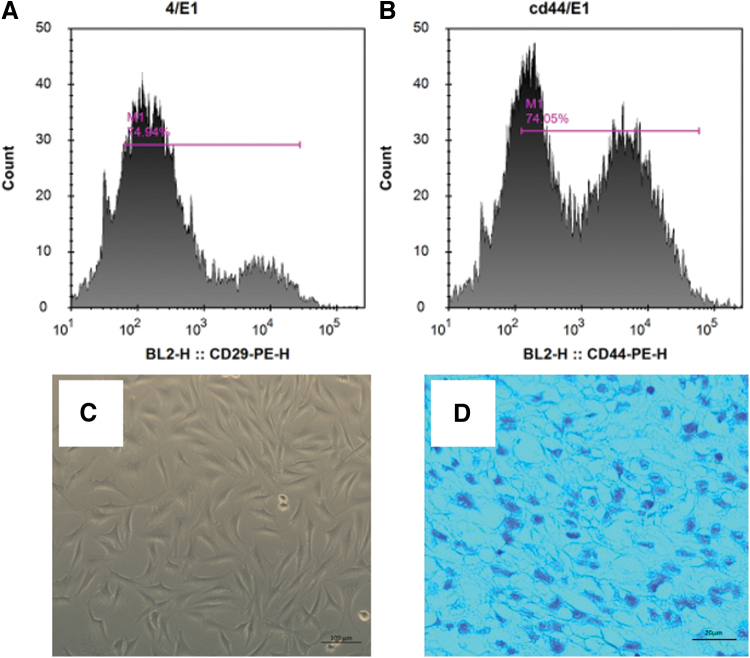
**(A, B)** Flow cytometry histograms for third-passage rADSCs showing CD29 and CD44 surface expression. **(C)** Microscopic image of 3rd-passage rADSCs. **(D)** Microscopic image of rADSCs after chondrogenic induction and Alcian Blue staining. rADSCs, rabbit adipose-derived stem cells. Color images are available online.

Flow cytometry analysis showed that CD29 and CD44 were positive at 74.94% and 74.05%, respectively, confirming the extracted cells as rADSCs (Fig. 7A–C). After 14 days of induction in a chondrogenic differentiation medium, rADSCs exhibited abundant protein secretion and intense blue staining of ECM rich in proteoglycans (Fig. 7D), indicating the capability of ADSCs for chondrogenic differentiation.

### The cytotoxicity of the ACM-SA scaffold

**FIG. 8.**
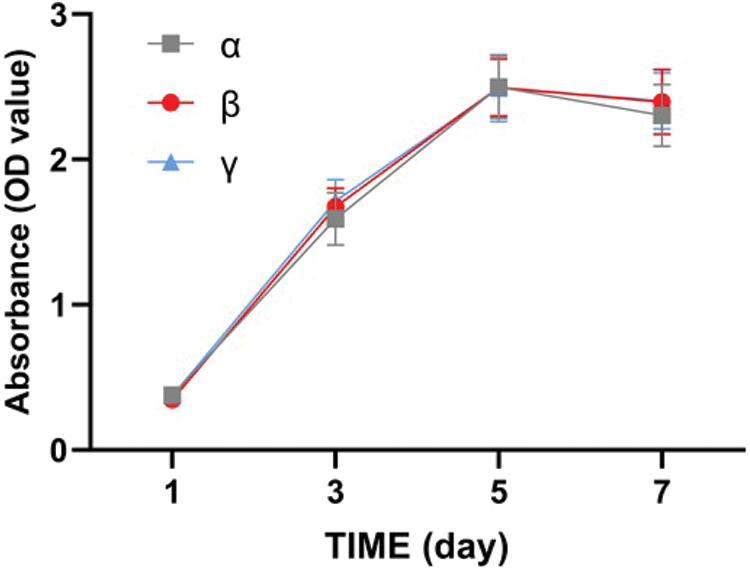
CCK-8 assay results for biotoxicity of ACM-SA scaffolds: α—Blank Control, β—1:9, γ—6:4 ratio, demonstrating excellent biocompatibility. Color images are available online.

After culturing cells in the scaffold extract for 7 days, all three cell groups exhibited robust proliferation. The OD values, measured using the CCK-8 assay, did not exhibit significant differences at different time points (*p* > 0.05), indicating excellent biocompatibility of the ACM-SA composite scaffolds (Fig. 8).

### The effect of the ACM-SA scaffold on the proliferation of rADSCs

**FIG. 9.**
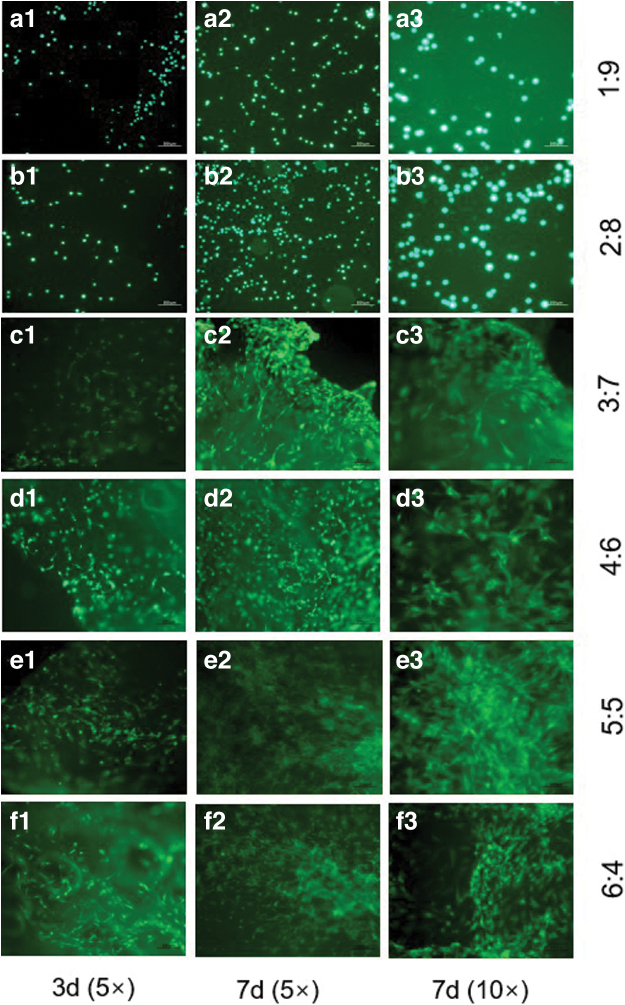
Images illustrating the adhesion and proliferation of rADSCs on ACM-SA scaffolds under chondrogenic conditions, with varied cell growth across different scaffold ratios. **(a1–a3)** Scaffolds with ACM:SA ratio of 1:9 showing initial cell adhesion. **(b1–b3)** Scaffolds with ACM:SA ratio of 2:8 showing initial cell distribution. **(c1–c3)** to **(f1–f3)** demonstrate cells adhering to the internal structure of the scaffolds with increasing fibrous-like growth observed from the third to the seventh day of culture, corresponding to ACM:SA ratios of 3:7, 4:6, 5:5, and 6:4, respectively. The ACM:SA (5:5) group, in particular, shows vigorous cell proliferation with the strongest fluorescence distribution observed under a microscope **(e2, e3)**. Color images are available online.

Except for the scaffolds with ACM: SA ratios of 1:9 and 2:8 (Fig. 9A1–3, B1–3), cells in all other groups adhered to the internal structure of the scaffolds and exhibited fibrous-like growth (Fig. 9C–F). On the seventh day, significant cell proliferation was observed in all groups within the scaffolds. In particular, the ACM:SA (5:5) group exhibited highly vigorous cell proliferation, with the strongest fluorescence distribution observed under a microscope (Fig. 9E2, 3).

**FIG. 10.**
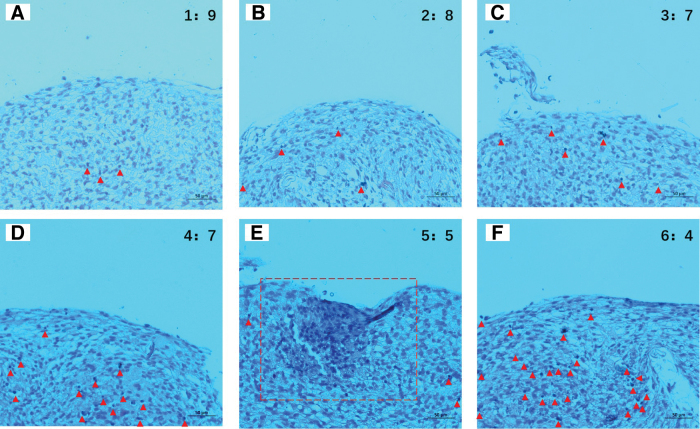
Alcian Blue staining of ACM-SA scaffolds, varying from ratios of 1:9 to 6:4, cocultured with rADSCs under chondrogenic conditions for three weeks. **(A)** Scaffolds with an ACM:SA ratio of 1:9. **(B)** Scaffolds with an ACM:SA ratio of 2:8. **(C)** Scaffolds with an ACM:SA ratio of 3:7. **(D)** Scaffolds with an ACM:SA ratio of 4:6. **(E)** Scaffolds with an ACM:SA ratio of 5:5, indicated by the red dashed box, showing a significantly higher amount of GAG precipitates. **(F)** Scaffolds with an ACM:SA ratio of 6:4. The deep blue clusters, marked by red arrows or within red boxes, represent GAG precipitates secreted by differentiated rADSCs, indicating chondrogenic activity. An increase in these GAG precipitates corresponds to enhanced cellular proliferation and secretory activity of the chondrocytes. GAG, glycosaminoglycan. Color images are available online.

After differentiating into chondrocytes, rADSCs synthesize and secrete glycosaminoglycans (GAGs), forming GAG precipitates, which are an important component of the ECM of chondrocytes. An increase in GAG content indicates an increase in the number and enhanced function of ADSCs that have proliferated and transformed into chondrocytes.^28–31^ GAGs, being a type of heteropolysaccharide, possess negatively charged carboxyl or sulfate groups and appear dark blue under Alcian Blue staining, as indicated by the red arrows and red boxes in the figure. As shown, in the six groups of scaffolds, the ACM:SA (5:5) group (Fig. 10E), indicated by the red dashed box, shows a significantly higher amount of GAG precipitates synthesized and secreted by the differentiated rADSCs compared to other groups.

**FIG. 11.**
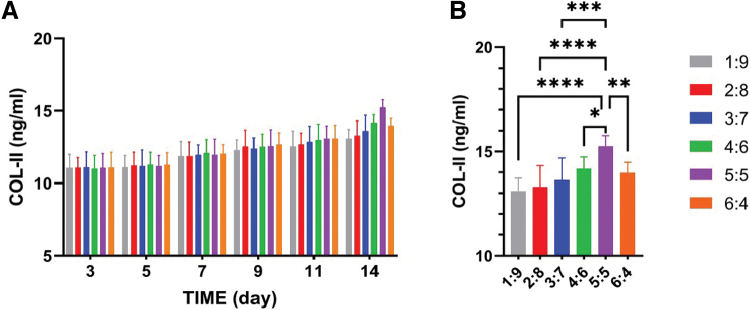
**(A)** ELISA-based quantitative analysis of free COL-II levels in ACM-SA scaffolds over time, showing significant differences in COL-II levels among different scaffold ratios. **(B)** Quantitative Analysis and Comparison of Free COL-II Levels in ACM-SA Scaffolds on Day 14. Statistical significance is indicated by *p < 0.05, **p < 0.01, ***p < 0.001, and ****p < 0.0001. COL-II, Collagen Type II; ELISA, enzyme-linked immunosorbent assay. Color images are available online.

As shown in Figure 11A, during the *in vitro* induction culture of the scaffolds on days 3, 5, 7, 9, 11, and 14, the content of free COL-II in the culture medium supernatant increased over time. On day 14, the levels of COL-II in the supernatant from the ACM:SA 5:5 groups were significantly higher than the other groups (Fig. 11B).

## Discussion

Based on *in vitro* experiments, it was observed that the ACM-SA scaffold with a ratio of 5:5 exhibited superior effectiveness in promoting the differentiation of rADSCs into chondrocytes compared to scaffolds with other ratios.

The ACM-SA scaffold promotes the differentiation and proliferation of rADSCs into chondrocytes. The degree of decellularization of ACM is crucial in allograft transplantation because residual chondrocytes in ACM can induce an immune reaction when they come into contact with the recipient's immune system, hindering the proliferation of rADSCs and their transformation into chondrocytes.^32^ ACM exhibits good biocompatibility and provides a favorable microenvironment for the transformation and proliferation of seeded cells.^33^ However, ACM alone has limitations due to its low viscosity and surface tension, making it unsuitable for 3D bioprinting and typically used as a gel material.^34,35^ These limitations can be overcome by combining ACM with SA, as the mixture can create stronger structures, and SA does not elicit an immune reaction during transplantation.^36^ The mechanical properties of the ACM-SA scaffold are also crucial.

When the proportion of SA in the ACM-SA hydrogel is below 30%, the printed structure cannot be formed. This is because SA can form insoluble and mechanically strong adhesives when crosslinked with Ca2+.^37^ If the proportion of SA is too low or insufficiently crosslinked with Ca2+, the scaffold becomes difficult to shape. ACM alone cannot solidify or form a 3D model after crosslinking with Ca2+. The porosity of the scaffold plays an important role in the differentiation of rADSCs.^38^ In this experiment, the 5:5 ratio of the ACM-SA scaffold showed the best results in promoting the transformation and proliferation of rADSCs. From the perspective of the mechanical properties of the scaffold, small pores favor the formation of hypoxic conditions, promoting the transformation, proliferation, and migration of rADSCs into chondrocytes.^39^

The degradation rate of the biomaterial scaffold should match the growth and healing rate of cartilage. Rapid degradation prevents the formation of the desired structure for new cartilage and can cause inflammation due to the phagocytosis of degraded byproducts by macrophages. Slow degradation hinders the growth of chondrocytes within the scaffold.^40–42^ In rabbit and human studies, reimplanted cartilage takes about 3 months to heal and stabilize,^43^ which aligns with the recovery time observed in patients undergoing cartilage repair surgery. In this experiment, the degradation rate of the ACM-SA scaffold with proportions ranging from 1:9 to 6:4 met the requirements. The increase in SA content in the ACM-SA scaffold led to a significant decrease in water absorption and swelling ratios. This is because SA crosslinks with Ca2+ to form CaCO_3_, which deposits on the surface of the scaffold, reducing porosity, water absorption, and swelling ratios.^44^

Subsequent cell immunofluorescence staining and quantification of free COL-II and confirmed that the 5:5 group with lower water absorption and swelling ratios exhibited the highest cell proliferation and secretion activity within the scaffold. This suggests that water absorption and swelling ratios are secondary factors influencing cell proliferation within the scaffold. Moreover, in clinical applications, especially in repairing specific shapes and sizes of cartilage defects, high swelling ratios can hinder the application of the scaffold. In clinical use, we expect the scaffold to have good compressive strength and sufficient hardness. In the compression performance tests conducted in this experiment, the 5:5 ratio scaffold had a low compression modulus and high hardness, remaining intact even when compressed to 60% strain. This is also related to the crosslinking of SA with Ca2+ to form CaCO_3_.

This study was conducted to perform a more comprehensive *in vitro* experimental examination of biomaterial scaffolds that can be used for the repair of auricular and nasal alar or septal cartilage defects. The strength required for the scaffold does not need to reach the level of articular cartilage. In future clinical applications, the scaffold only needs to guide the growth of seeded cells according to their shape and provide support for overlying skin. The cytotoxicity, tissue compatibility, and adhesion properties of the biomaterial scaffold are crucial for its application.^45^ SA and ACM themselves are nontoxic to cells,^46–48^ which is supported by the results of the cytotoxicity experiments conducted in this study. As shown in the figure, as the proportion of ACM in the scaffold increases, the adhesion rate of rADSCs to the scaffold gradually improves, enhancing cell viability and secretion function.

It can be inferred that in the ACM-SA scaffold, the crosslinking of SA with Ca2+ primarily affects the formation and mechanical properties of the scaffold, while ACM plays a significant role in the differentiation and proliferation of rADSCs. Only a suitable ratio of these two components can simultaneously meet the mechanical requirements and biological performance of the scaffold.

Compared to traditional nonbiological scaffolds, ACM-SA scaffolds retain the composition, biomechanics, and structural characteristics of the native ECM, naturally mimicking the complex extracellular microenvironment. They exhibit high bioactivity and lower immunogenicity, while also having good biodegradability. Decellularized matrix scaffolds, serving as cell-guiding matrices, aid in promoting the proliferation, factor secretion, and differentiation of stem cells in tissue-specific manners.^49,50^ This is not to say that nonbiological scaffolds are inferior; nonbiological scaffolds, such as synthetic polymer scaffolds (e.g., polylactic acid, polyglycolide, polycaprolactone, polyvinyl alcohol), are easier to control and standardize during production, generally cost less, and are easier to produce on a large scale. The physical and chemical properties of nonbiological scaffolds can be precisely adjusted to meet specific application needs, but their use is limited due to restricted bioactivity and the potential for long-term tissue response or chronic inflammation caused by biodegradability.

Similar to the studies by Gonzalez-Fernandez et al., SA gels alone lack endogenous motifs for cell adhesion.^51^ However, the addition of decellularized matrix or collagen to SA significantly promotes cell adhesion and proliferation, and increases as the proportion of decellularized matrix or collagen rises within a certain range.^52–54^ From this, we can speculate that when repairing different tissues in clinical treatments, the corresponding tissues can be made into decellularized matrices and then mixed with SA to prepare as 3D printing materials, and then mixed into different ratios of bioink according to required characteristics. The currently studied decellularized matrix/SA mixed inks all exhibit commendable bioactivity and biosafety, as well as adjustable physical properties.^52,53,55–58^

In future research, we will explore the use of suitable growth factors to promote the differentiation of rADSCs into chondrocytes and facilitate the rapid growth of mature cartilage cells. In addition, we may investigate the addition of certain substances to inhibit the formation of scar tissue.

## Conclusions

A biomaterial scaffold should possess excellent biocompatibility and cell adhesion properties to promote the directed differentiation and proliferation of seeded cells. In addition, it should exhibit mechanical properties, including appropriate porosity, mechanical strength, swelling ratio, and degradation ratio, tailored to specific applications.^59–61^ In the fabrication of biomaterial scaffolds, the materials used should be readily available, cost-effective, and easy to manufacture. Through this study, we conducted a comprehensive analysis of the biological and mechanical properties of ACM-SA scaffolds with varying ratios, leading to the following conclusions: when the proportion of SA in the ACM-SA scaffolds was <30%, the printed structure failed to form. The ACM-SA scaffolds in proportions from 1:9 to 6:4 showed no significant cytotoxicity, among which the 5:5 proportion of ACM-SA scaffold was superior in terms of adhesiveness and promoting cell proliferation and differentiation.

Although a higher proportion of SA can provide greater mechanical strength, it also significantly increases the swelling ratio and reduces cell proliferation capabilities. Overall, the 5:5 proportion of ACM-SA scaffold demonstrated a more desirable biological and physical performance.

## Data Availability

The data used to support the findings of this study are available from the corresponding author upon request.
